# Purification, structural characterization, and bioactive properties of exopolysaccharides from *Saccharomyces cerevisiae* HD-01

**DOI:** 10.3389/fbioe.2024.1455708

**Published:** 2024-08-22

**Authors:** Ruoxi Yang, Lina Liu, Dongni Gao, Dan Zhao

**Affiliations:** Engineering Research Center of Agricultural Microbiology Technology, Ministry of Education, Heilongjiang Provincial Key Laboratory of Plant Genetic Engineering and Biological Fermentation Engineering for Cold Region, Key Laboratory of Microbiology, College of Heilongjiang Province, School of Life Sciences, Heilongjiang University, Harbin, China

**Keywords:** *Saccharomyces cerevisiae*, exopolysaccharides, identification, characterization, immunomodulation

## Abstract

Exopolysaccharides (EPSs), which show excellent biological activities, like anti-tumor, immune regulation, and anti-oxidation activities, have gained widespread attention. In this study, an EPS-producing *Saccharomyces cerevisiae* HD-01 was identified based on 18S rDNA sequence analysis and an API 20C test. The purified HD-01 EPS was obtained by gel filtration chromatography. High-performance liquid chromatography (HPLC), gel permeation chromatography (GPC), Fourier transform infrared spectroscopy (FT-IR), and nuclear magnetic resonance (NMR) revealed that it was a heteropolysaccharide composed of *α*-1 (38.3%), *α*-1, 2 (17.5%), *α*-1, 6 (14.8%)-linked mannose and *α*-1, 2, 3, 6 (24.3%), *α*-1 (3.3%), *β*-1, 4 (1.8%)-linked glucose. Chemical composition and elemental analysis indicated the existence of sulfation modifications. A scanning electron microscope (SEM) and an atomic force microscope (AFM) revealed that it exhibited a flaky structure with thorn-like protrusions on the three-dimensional surface. X-ray diffraction (XRD) revealed that it was an amorphous non-crystalline substance. HD-01 EPS had great thermostability; probiotic properties; strong antioxidant properties to DPPH, ABTS, and hydroxyl; and good reducing power. The MTT, NO, and neutral red assays demonstrated that it had a great immunomodulatory effect on macrophages RAW264.7. All results suggested that the HD-01 EPS had the potential to be applied in the food and pharmaceutical fields.

## 1 Introduction

Exopolysaccharides (EPSs), which are safe and degradable, are macromolecular substances secreted out of cells during the metabolism of microorganisms (Min et al., 2018). Based on the composition, EPSs have been classified into two groups: homopolysaccharides (HoPSs) composed of one type of sugar and heteropolysaccharides (HePSs) composed of two or more types of sugar according to the chemical composition (Rahbar et al., 2021). It has been reported that EPS has a multitude of benefits across various fields (Prabhu and Kannan, 2024). From the perspective of function, the biological activity of EPS can be applied in the food and pharmaceutical fields (Zhou et al., 2019). Lactic acid bacteria EPS is commonly employed as a natural additive to enhance the texture of food and biological materials and boost the biological activity of medications (Zhang et al., 2024). Yeast, which has shown to play an important role in probiotics used to treat diseases and improve immunity, also possesses the capability of producing EPSs. EPSs produced by yeast are easier to separate due to their short generation time and have become one of the hotspots in industrial microbial research (Liu et al., 2022). Closely related to the structure, yeast EPSs have been reported to show biological beneficial activities, like anti-tumor, cholesterol-lowering, immune regulation, and anti-oxidation activities (Pintado et al., 2019; Videva et al., 2019). Meanwhile, yeast EPSs are used as stabilizers, thickeners, and bioflocculants (Ragavan and Das, 2019) and have gained widespread attention in the food and pharmaceutical industries.

It is reported that EPS-producing yeasts include *Saccharomyces*, *Pichia*, *Candida*, *Rhodotorula*, *Sporobolomyces*, *Bullera*, etc. (van Bogaert et al., 2009). *Saccharomyces cerevisiae* is a model strain used as an important microorganism in the alcohol brewing and bread baking industries. EPSs have various structures. Differences in monosaccharide compositions, glycosidic bonds, functional groups, and molecular weight (Mw) may result in specific functional characteristics, such as antioxidant, immunomodulation, and antitumor activities (Liu et al., 2022). EPSs could enhance the rheological properties, microstructural stability, and water-holding capacity; hence, they are commonly known as emulsifiers and gelling agents in the food industry (Li et al., 2022). In addition to their incorporation into food products, EPSs also exhibit remarkable immunomodulatory effects (Zhang et al., 2024). Therefore, studying the structural–activity relationship could help broaden the range of applications of EPSs (Du et al., 2022a; Du et al., 2022b). However, there are few studies focused on *S. cerevisiae* EPS and its structure, properties, and functional characteristics.

In this study, an EPS-producing strain, *S. cerevisiae* HD-01, previously isolated from the soil beside the lees and preserved in the laboratory was identified based on morphological observation, API 20C, and 18S rDNA. EPS was separated and purified. Its chemical composition, monosaccharide composition, Mw, glycosidic bond, crystal structure, and glycopeptide chain were determined. The microstructure of EPS was characterized by scanning electron microscopy (SEM) and atomic force micrograph (AFM). The water solubility index (WSI), water holding capacity (WHC), intrinsic viscosity, thermodynamic properties, contact angle, probiotics, and antioxidant properties of EPS were revealed to explore its potential application. The immunomodulatory effect of EPS on mononuclear macrophages RAW264.7 was also evaluated. This study provided a theoretical foundation for the structure–activity relationship and the application potential of HD-01 EPS in various fields.

## 2 Materials and methods

### 2.1 Yeast strain and cultural conditions

The strain HD-01 was preserved in the Key Laboratory of Microbiology, Heilongjiang University. The YPD medium composed of glucose 2% (w/v), tryptone 2% (w/v), and yeast extract 1% (w/v) at 160 rpm for 24 h at 30°C was used for the activation and preservation of HD-01. The EPS-producing medium with sucrose 7% (w/v), NH_4_SO_4_ 0.2% (w/v), KH_2_PO_4_ 0.2% (w/v), yeast extract 0.58% (w/v), and CaCl_2_ 0.03% (w/v) at pH 5.3, 30°C, and 160 rpm for 144 h was used to produce HD-01 EPS.

### 2.2 Chemicals and reagents

The API 20C reagent strip (REF20210) was purchased from France Merieux Co., LTD. The Yeast Genomic DNA Extraction Kit (DP307-02) was purchased from Beijing Tiangen Biochemical Technology Co. LTD. The MTT Cell Proliferation and Cytotoxicity Assay Kit (C009S) was purchased from Biyuntian Biotechnology Co., LTD. The NO Assay Kit (S0021S) was purchased from Biyuntian Biotechnology Co., LTD. The ELISA kit was purchased from Shanghai Enzyme Linked Biotechnology Co., LTD. Dulbecco’s modified Eagle’s Medium (DMEM) was purchased from Cytiva; the penicillin–streptomycin solution, Fetal Bovine Serum (FBS), and phosphate-buffered saline (PBS) were purchased from HyClone. Vitamin C (Vc) (A8100) was purchased from Solarbio. Phenol and absolute ethanol were all domestically produced and were analytically pure.

### 2.3 Strain identification

#### 2.3.1 Physiological and biochemical identification

According to the method of Schuffenecker et al. (1993), the API 20C reagent strip was used to determine the fermentation of carbohydrates produced by the HD-01. Cultured on the YPD agar medium, colonies were picked and dissolved in 0.85% (w/v) sodium chloride solution. The 2 McFarland (*OD*_*550 nm*_ = 0.5) HD-01 suspension was prepared. The HD-01 suspension was added to an ampoule of API 20C and mixed. Incubated at 30°C for 48 h and 72 h, the characteristics were identified by referring to the analysis chart index or identification software.

#### 2.3.2 Identification with 18S rDNA

The HD-01 was cultured on the YPD medium at 30°C for 12 h. The total DNA was extracted using the Yeast Genomic DNA Extraction Kit. The upstream primer 5′ GTAGT CATAT GCTTG TCTC 3′ and the downstream primer 5′ GCATC ACAGA CCTGT TATTG CCTC 3′ were used for PCR amplification (Britta et al., 2016). Compared with the NCBI database, a phylogenetic tree was constructed.

### 2.4 Extraction and purification of HD-01 EPS

The purification was determined based on the study of Zhao et al. (2019). The HD-01 (1% (v/v)) was cultured into EPS-producing medium at 30°C and 160 rpm for 144 h. It was the then centrifuged (8,000 × g, 45 min, 4°C) to remove pellets. Pre-cooled 95% ethanol was added to the supernatant, kept overnight at 4°C, and centrifuged. It was then dissolved in deionized water. TCA (10%) was added to remove protein and centrifuged (8,000× g, 45 min, 4°C). Precipitation with 95% pre-cooled ethanol was carried out. It was then dissolved in deionized water and transferred to a dialysis bag (Mw cut-off, 14 kDa) at 4°C. It was purified by gel filtration chromatography (Sephadex G-100), followed by lyophilization. The final product was purified EPS (P-EPS).

### 2.5 Purity and monosaccharide composition of HD-01 EPS

The purity of EPS was identified in the wavelength range of 190–400 nm via an ultraviolet spectrophotometer (SP-1920UV, Spectrum Instruments, Shanghai, China).

The monosaccharide composition was analyzed via high-performance liquid chromatography (HPLC) (Waters, United States), referring to the method of Du et al. (2017). EPS (2 mg) was mixed with the anhydrous methanol solution containing 1 mol/L HCl and 2 mol/L trifluoroacetic acid (TFA). Hydrolysis was performed at 120°C for 1 h. Derivatization was performed with 1-phenyl-3-methyl-5-pyrazolone (PMP). The PMP-derivatized EPS (10 μL) was injected into the detector (1.0 mL/min), and the UV absorbance was measured at 245 nm.

### 2.6 Determination of Mw of HD-01 EPS

The Mw was measured via gel permeation chromatography (GPC), referring to the previously reported method (Du et al., 2022a). A measure of 2 mg/mL EPS was injected. The chromatographic column was Shodex OH pak SB-806 (8.0 mm × 300 mm). NaNO_3_ (0.1 M) was used as the mobile phase. Detection was carried out using a differential multi-angle laser light scattering device (DAWN EOS, Wyatt, Shanghai, China).

### 2.7 Chemical composition analysis of HD-01 EPS

The contents of carbon, hydrogen, nitrogen, and sulfur in P-EPS were determined using an elemental analyzer (Seedevi et al., 2015). The EPS was put into the element analyzer, helium was blown in, and the CHNS mode was selected. The element content in the P-EPS was calculated with the standard curve.

The phenol–sulfuric acid method (Dubois et al., 1980), Bradford protein assay (Bradford, 1976) (bovine serum albumin as a standard), and CaCl_2_–gelatin method (Dodgson and Price, 1962) (K_2_SO_4_ as a standard) were used to determine the total sugar, protein, and sulfuric acid group in HD-01 EPS, respectively.

### 2.8 Morphological analysis of HD-01 EPS

Surface morphology of HD-01 P-EPS was observed by SEM (S-4800, Hitachi, Japan). The sample was adhered to the table and coated with a conductive gold layer. The SEM was conducted under 10-kV acceleration voltage at different magnification rates (×200 and ×1,000) (Liu et al., 2022).

According to the method of Zhao et al. (2021), the macro morphology of HD-01 P-EPS in the aqueous solution was determined via AFM (NT-MDT, Ntegra spectra, Russia). It was then dissolved in deionized water to the concentration of 1 mg/mL. After diluting 5 μL of P-EPS solution to the concentration of 10 μg/mL, it was spread onto mica and dried. The response frequency, elastic coefficient, and amplitude were measured at 340.7 kHz, 0.35 N/m, and 0.52 V, respectively.

### 2.9 X-ray diffraction (XRD) analysis of HD-01 EPS

The P-EPS powder was added to the X-ray diffractometer (SmartLab, Japan). The diffraction pattern was obtained (2°/min, 5°–90°).

### 2.10 Fourier transform infrared (FT-IR) spectroscopy analysis of HD-01 EPS

FT-IR of HD-01 P-EPS was measured via a BIO-RAD IR spectrometer (FTS3000, Bruker, Karlsruhe, Germany) to obtain the functional group components (Du et al., 2017). P-EPS was mixed with KBr powder (1:100) in thin slices (1 nm). The sample was scanned by FT-IR at 4,000–400 cm^−1^ with a detector resolution of 1 cm^−1^.

### 2.11 Nuclear magnetic resonance (NMR) analysis of HD-01 EPS

According to the method of Zhao et al. (2019), the P-EPS powder (30 mg) was dissolved in D_2_O in an NMR tube. ^1^H-NMR, ^13^C-NMR, COSY, and HSQC were obtained by NMR spectroscopy (Avance III, Bruker, United States). The relaxation wait time was 1.5 s. D_2_O was used as the internal standard. Data were analyzed using MestReNova software.

### 2.12 Glycosidic bonding of HD-01 EPS

Methylation was used to analyze glycosidic bond types (Oluwa, 2020). It was completely dissolved in anhydrous dimethyl sulfoxide (DMSO) and filled with N_2_. NaOH–DMSO (0.5 mL) was then added. An equal volume of dichloromethane was added for extraction (30 min). The methylated EPS had a significant hydroxyl absorption peak at 3,400 cm^−1^ detected by infrared spectroscopy. No absorption peak of the base product was observed, proving that the EPS was completely methylated. The methylated EPS was hydrolyzed, reduced, and acetylated. After being dissolved with 1 mL dichloromethane and filtered by the filter membrane, gas chromatography–mass spectrometry (GC–MS) analysis was performed.

### 2.13 Carbohydrate–peptide linkage analysis

The connection between carbohydrates and peptide bonds in HD-01 P-EPS was determined via a *β*-elimination reaction (Manhivi et al., 2018). The P-EPS powder (10 mg) was dissolved in 1 mol/L NaBH_4_–NaOH solution and placed in a 45°C water bath for 24 h. The absorbance of the mixed solution within 190–400 nm was measured by UV-vis spectrophotometry, with the alkali-treated sample solution as the control.

### 2.14 Determination of intrinsic viscosity

The intrinsic viscosity [*η*] (1/[c], mL/g) of HD-01 P-EPS under 25°C and 35°C were measured using an Euclidean viscometer (1835-3, Fangqi Instruments, Ltd., Shanghai, China), respectively. The outflow time of the EPS solution (t) and solvent (t_0_) were recorded. According to the following formula, the relative viscosity (*η*_*r*_), specific viscosity (*η*_*sp*_), *η*_*sp*_/*c*, and *lnη*_*r*_/*c* were calculated. The EPS solution concentration (c) and *η*_*sp*_/*c* and *lnη*_*r*_/*c* were taken as the abscissa and ordinate, respectively. The intrinsic viscosity [*η*] was calculated. Independent triplicates were performed in each group (Huggins, 1942).

$$\eta_{r}=\frac{t}{t_{0}},$$

$$\eta_{sp}=\eta_{r}-1,$$

$$\text{Huggins formula }{\frac{\eta_{sp}}{c}}_{=}\left[\eta \right]+k^{\prime }{\left[\eta \right]}^{2}c,$$

$$\text{Kraemer formula }\ln\frac{\eta_{r}}{c}=\left[\eta \right]+k^{\prime \prime }{\left[\eta \right]}^{2}c.$$

### 2.15 WSI assay

The HD-01 P-EPS WSI was determined referring to Zhao et al. (2019). It was fully dissolved in 0.5 mL deionized water, weighed (M_1_), and centrifuged (4,000× g, 10 min). The pellet was lyophilized and weighed (M_2_). The WSI was calculated as follows:

$$\text{WSI }\left(\%\right)=\left[\left(M_{1}‐M_{2}\right)/M_{1}\right]\times 100.$$

### 2.16 WHC assay

According to the method of Yang et al. (2018), P-EPS (W_1_, 45 mg) was dissolved in deionized water (5 mL) and stirred at 40°C for 40 min. Followed by centrifuging (4,000×g, 10 min), the pellet was lyophilized and weighed (M_2_). The WHC was calculated as follows:

$$\text{WHC }\left(\%\right)=\frac{W_{2}}{W_{1}}\times 100.$$

### 2.17 Thermal properties

The thermal properties of HD-01 P-EPS and the change in the aggregation state with temperature were tested using a Netzsch integrated thermal analyzer (STA449F3, Germany). Differential thermal scanning analysis (DSC), thermogravimetric analysis (TGA), and differential thermogravimetric analysis (DTG) were performed. P-EPS (4 mg) was put into an Al_2_O_3_ crucible with Ar and the linear heating rate of 10°C/min from 25°C to 800°C (Liu et al., 2022).

### 2.18 Contact angle test

The contact angle was determined according to the study of Jiang et al. (2020), with a slight modification. The strain HD-01 (2% (v/v)) was inoculated into 20 mL/50 mL YPD and EPS-producing medium. They were cultured at 30°C for 24 h and 144 h, respectively. Following centrifuge at 8,000 × g for 25 min, the supernatant was filtered using a 0.22-μm filter membrane. The data were captured by contact angle analyzer (JY-82B, Chengde Dingsheng Test Machine Equipment Co., Ltd., China).

### 2.19 Probiotics of HD-01 EPS

Six probiotics (*Lactobacillus plantarum* subsp. CICC 6076, *Lactobacillus delbrueckii* subsp. CICC 6077, *Lactobacillus acidophilus* QY01, *Lactobacillus casei* QY02, *Bifidobacterium adolescentis* QY03, and *Lactobacillus paracei* HD1.7) were cultured in 20 mL/50 mL MRS medium at 30°C for 12 h, respectively. The six probiotics [1% (v/v)] were inoculated into 100 mL/250 mL MRS medium supplemented with commercial prebiotics [inulin, mannooligosaccharide (MOS), galactose oligosaccharide (GOS), and fructose oligosaccharide (FOS)], glucose, and 1% EPS, respectively. They were cultured at 30°C for 48 h. *OD*_*600 nm*_ was determined. The proliferative effects of EPS and prebiotics were compared.

### 2.20 *In vitro* antioxidant activity assay of HD-01 EPS

#### 2.20.1 Scavenging assay of 1,1-diphenyl-2-picrylhydrazyl (DPPH) radical

Based on the method of Liang et al. (2016) and appropriate improvement, the DPPH radical scavenging test was performed. The different concentrations of HD-01 EPS (2 mL 0–4 mg/mL) were added to the DPPH solution (2 mL 0.2 mmol/L), respectively, and reacted for 30 min in the dark. The absorbance at *OD*_*517 nm*_ was determined with Vc as the control. The DPPH radical scavenging activity was determined as follows:

$$\text{Scavenging activity }\left(\%\right)=\left[1‐\left(A_{1}‐A_{2}\right)/A_{0}\right]\times 100.$$

Note: A_0_ was the absorbance value of the deionized water. A_1_ was the absorbance value of the EPS with the DPPH radical. A_2_ was the absorbance value of DPPH with deionized water.

#### 2.20.2 Scavenging activity of the 2,2′-azinobis-bis(3-ethylbenzthiazoline-6-sulphonate) (ABTS^+^) radical

The determination of the ABTS^+^ radical scavenging ability refers to the study of Wootton-Beard et al. (2011). ABTS^+^ (7 mmol/L) was mixed with potassium persulfate aqueous solution (2.45 mmol/L) in equal volume. It was then kept at room temperature for 16 h and diluted with 0.2 mol/L phosphate buffer (pH 7.4). P-EPS solution (0.2 mL, 0–4 mg/mL) was mixed with 4 mL ABTS^+^ solution, respectively, and reacted at room temperature for 6 min. *OD*_*734 nm*_ was determined with Vc as the control. The ABTS^+^ radical scavenging activity was determined according to the formula:

$$\text{Scavenging activity }\left(\%\right)=\left[1‐\left(A_{1}‐A_{2}\right)/A_{0}\right]\times 100.$$

Note: A_0_ was the absorbance value of the deionized water. A_1_ was the absorbance value of the EPS with the ABTS^+^ radical. A_2_ was the absorbance of ABTS^+^ with deionized water.

#### 2.20.3 Scavenging assay of the hydroxyl radical

Hydroxyl radical scavenging activity is based on the study of Wang et al. (2019a). The HD-01 P-EPS solution (1 mL) with different concentrations (0–4 mg/mL), 9 mmol/L FeSO_4_ solution (1 mL), 9 mmol/L salicylic acid–ethanol solution (1 mL), and 9 mmol/L H_2_O_2_ solution (1 mL) were mixed and reacted at 37°C for 40 min. The absorbance value of *OD*_*510 nm*_ was measured with Vc as the positive control. The hydroxyl radical scavenging capacity of the EPS was calculated:

$$\text{Scavenging activity }\left(\%\right)=\left[1‐\left(A_{1}‐A_{2}\right)/A_{0}\right]\times 100.$$

Note: A_0_ was the absorbance value of deionized water. A_1_ was the absorbance value of the EPS with ·OH^−^. A_2_ was the absorbance of ·OH^−^ with deionized water.

#### 2.20.4 Reducing power assay

Reducing power of HD-01 P-EPS was determined referring to Zhang et al. (2010). EPS solution (1 mL, 0–4 mg/mL) was mixed with 2.5 mL phosphate buffer (0.2 mol/L, pH 6.6) with 2.5 mL 1% (W/V) potassium ferricyanide solution and reacted at 50°C for 20 min. TCA [2.5 mL, 10% (w/v)] was added and centrifuged (4,000 × g, 30 min). FeCl_3_ [0.5 mL 0.1% (W/V)] and 2.5 mL deionized water were added to the supernatant and reacted for 10 min. The absorbance value of *OD*_*700*_ _nm_ of the reaction system was measured with Vc as the positive control. The experiment was repeated three times.

### 2.21 Immunomodulatory activity assay on RAW264.7 cells

#### 2.21.1 Cell lines and cell culture

Mononuclear macrophage RAW264.7 cells purchased from the Harbin Veterinary Research Institute (Harbin, China) were resuscitated and cultured in a DMEM high-glucose medium containing 10% fetal bovine serum and penicillin and streptomycin double antibodies (100 μL). Cultivated in a 5% CO_2_ incubator at 37°C, the cells were subcultured on 3–4 generations for experiments (Yang et al., 2019).

#### 2.21.2 Assay for cell viability

The proliferation effect of HD-01 P-EPS on RAW264.7 cells was determined using the MTT Kit. Cultured to the logarithmic growth phase, the cells were inoculated into 96-well plates. Suspension (100 μL) was added to each. Filled with PBS, the 96-well plate was cultured in a CO_2_ incubator at 37°C for 24 h. After adhering to the wall, the medium was removed and washed with PBS. The control group (Ac) was medium cultured cells. LPS (100 μL, 1%) was added to the positive control group, and 100 µL HD-01 P-EPS solution of different concentrations (10, 50, 100, and 200 μg/mL) was added to experimental groups (Ae), with medium cultured cells as the control group (Ac). Each group was repeated six times. It was cultured in a 5% CO_2_ incubator at 37°C for 24 h. MTT solution (100 μL, 1 mg/mL) was then added. After culturing for 4 h, the supernatant was removed. DMSO (150 µL) was added and allowed to react at dark for 10 min. The value of *OD*_*490 nm*_ was determined. The survival rate of RAW264.7 cells was calculated as follows:

$$\text{Cell viability }\left(\%\right)=\frac{\text{Ae}}{\text{Ac}}\times 100.$$

#### 2.21.3 NO assay

The NO Assay kit was used to determine NO production. After culture in a 5% CO_2_ incubator at 37°C for 24 h, the medium was removed and washed with PBS. The positive control group added with LPS (1 μg/mL) was cultured in complete medium, and the experimental groups were added with the P-EPS solution of different concentrations (10, 50, 100, and 200 μg/mL) with cells cultured in complete medium as the control. Each was repeated six times. It was then cultured for 24 h and centrifuged (1,000×g, 15 min). The supernatant was added to a 96-well plate. Griess A and B reagents (50 µL) were added, respectively. The value of *OD*_*540 nm*_ was measured. NO production of RAW264.7 cells was calculated according to the standard curve of NaNO_2_.

#### 2.21.4 Phagocytic assay

The effect of HD-01 P-EPS on the phagocytosis of RAW264.7 cells was detected by the neutral red method (Zhang et al., 2017). RAW264.7 cells (100 µL) were cultured in a 5% CO_2_ incubator at 37°C for 24 h. The medium was removed and washed with PBS. The experimental group (Ae) was cultured with P-EPS solution of different concentrations, with cell culture medium as the control (Ac) and LPS (1 μg/mL) added as the positive control. The medium was removed and washed with PBS three times. The neutral red solution [100 μL, 0.075% (m/v)] was added and cultured for 1 h. Then, the supernatant was removed, and the cell lysate was added. It was cultured for 4 h. The value of *OD*_*540 nm*_ was measured. The phagocytosis rate of RAW264.7 cells was calculated according to the following formula:

$$\text{Phagocytosis rate }\left(\%\right)=\frac{\text{Ae}}{\text{Ac}}\times 100.$$

#### 2.21.5 Determination of IL-6, IL-8, IL-1*β*, IL-10, MCP-1, and TNF-*α*

Cultured to the logarithmic stage, RAW264.7 cells were inoculated into 96-well cell culture plates (100 μL). After adhering, the supernatant was removed. The HD-01 EPS solution (100 μL) with different concentrations was added. The cell culture medium (100 μL) was added to the blank control group, and LPS (100 μL, 10 μg/mL) was added to the positive control group. Each was repeated three times. Kept in a 5% CO_2_ cell incubator at 37°C for 24 h, the supernatant (10 μL) was collected, and the release of cytokines was determined according to the instructions of the ELISA kit.

### 2.22 Statistical analysis

All results were obtained in independent triplicates and presented as the mean ± standard deviation. JMP statistical software (9.0.2, SAS Institute Inc., United States) was used for statistical analysis, with a significance level set at *p* < 0.05 for one-way analysis of variance (ANOVA). Additional statistical analysis was performed using Origin2022 software (OriginLab Corporation, Northampton, MA, United States). MestReNova software (MestReNova x64-14.2.1, Mestrelab Research, Santiago de Compostela, Spain) was used for NMR analysis.

## 3 Results and discussion

### 3.1 Strain identification

**FIGURE 1:**
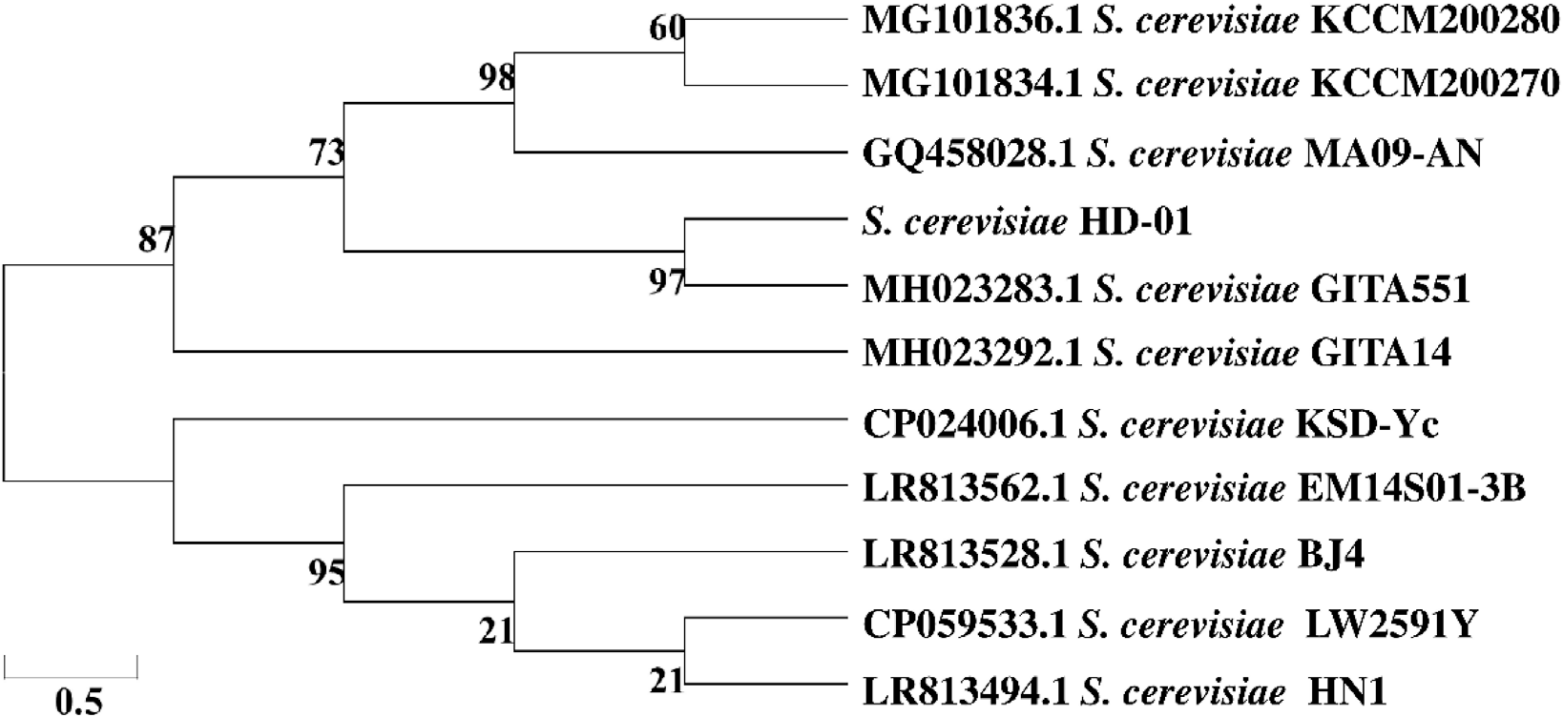
Phylogenetic tree of *S. cerevisiae* HD-01 based on the neighbor-joining method.

API 20C was an identification system containing biochemical reaction tests and specific databases. The identification was performed based on %ID and T-value, showing that HD-01 was consistent with *S. cerevisiae* (%ID: 98.7%; T-value: 1.0). The 18S rDNA sequencing results revealed the HD-01 sequence length was 1,377 bp. It was submitted to the NCBI database, and the GenBank accession number (MW295845) was obtained. The phylogenetic tree is displayed in Figure 1, showing 97% homology with *S. cerevisiae* GITA551 (MH023283.1), which was in accordance with the result of API 20C. The HD-01 was identified as *S. cerevisiae*.

### 3.2 Purification and monosaccharide composition

**FIGURE 2:**
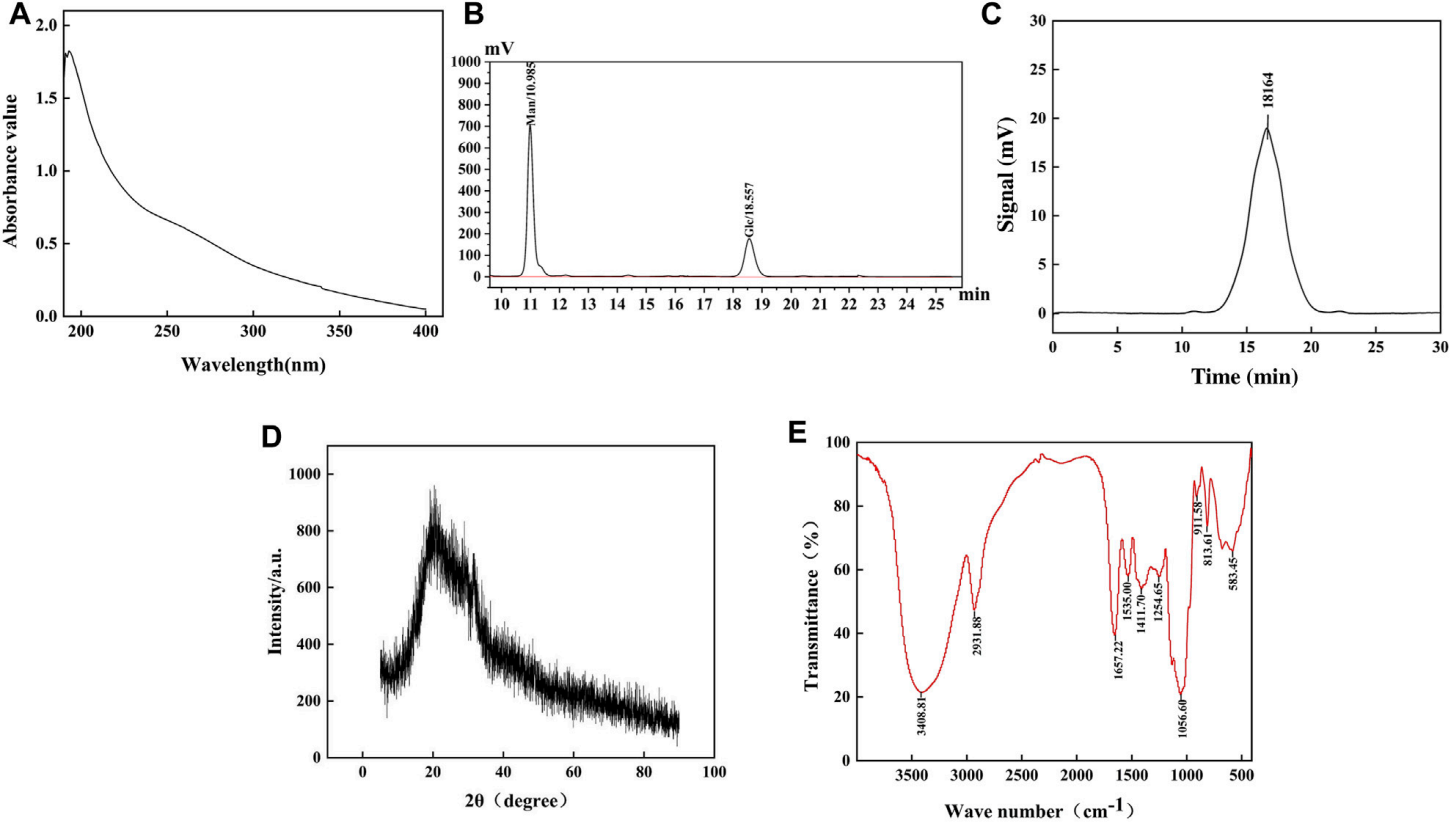
Ultraviolet spectrum **(A)**, HPLC **(B)**, GPC **(C)**, XRD **(D)**, and FT-IR **(E)** of *S. cerevisiae* HD-01 EPS.

The absence of absorption peaks at 260 nm and 280 nm confirmed that there were no nucleic acids and protein contaminations, as depicted in Figure 2A. The monosaccharide composition of HD-01 EPS was elucidated via HPLC (Figure 2B), showing that it was mainly composed of mannose (68.66%) and glucose (27.32%). Amer and Mubarak (2013) found that *S. cerevisiae* EPS was mainly arabinose and ribose, which was inconsistent with the results of this study. This might be related to the components of the medium (Gientka et al., 2015). GC–MS revealed that *Rhodosporidium babjevae* EPS was composed of mannose and glucose (Seveiri et al., 2020). *Papiliotrema flavescens* EPS was composed of mannose (45.4%), xylose (45.4%), and a small amount of glucose (9.1%) (Oluwa, 2020). *Cryptococcus laurentii* AL_100_ EPS was composed of arabinose (61.1%) and mannose (15.0%) (Pavlova et al., 2011). These results indicated that yeast EPS was mostly heteropolysaccharide.

Previous studies have shown that the biological characteristics of yeast EPS were related to the monosaccharide composition. Various monosaccharides had diverse functions. *Rhodotorula glutinis* EPS, composed of mannose, glucose, and arabinose, had antioxidant, antiviral, and antitumor activities (Ghada et al., 2012). EPS containing high levels of glucose and mannose had a great immune-stimulating effect, which might be because glucose could be an immune adjuvant for anti-tumor treatment, and there was a mannose receptor on the surface of the macrophage (Zhang et al., 2023). This suggested that EPSs rich in glucose and mannose possessed significant immunomodulatory capabilities. The monosaccharide composition of EPS derived from various microbial sources can influence their biological activities. Mannose-rich EPS from *Bacillus amyloliquefacien*s DMBA-K4 were able to alleviate colitis symptoms in mice by modulating gut microbiota (Kuang et al., 2020). Glucomannan-type EPS from *Bifidobacterium bifidum* enhanced NO secretion in RAW 264.7 cells (Kang et al., 2023).

### 3.3 Mw analysis of EPS

The Mw of HD-01 EPS could demonstrate the relationship between the structure and function. A symmetrical narrow peak could be observed when the retention was 15.3 min, indicating the great homogeneity (Figure 2C). The Mw of HD-01 EPS was 1.8164 × 10^4^ Da, which was lower than most yeast EPS, such as *Rhodotorula mucilaginosa* CICC 33013 EPS (7.125 × 10^4^ Da) (Ma et al., 2017). EPS with low Mw had great anti-tumor and antioxidant capacities. *Pestalotia* sp. 815 EPS had large Mw (greater than 2 × 10^6^), resulting in weak antitumor activity in rats, and low Mw of 4.7 × 10^5^ exhibited remarkable antitumor activities against mouse-implanted tumors (Misaki et al., 1984). A study has shown that the antioxidant capacity might be related to the number of hemiacetal hydroxyl groups, and lower Mw EPS may present a higher concentration of hemiacetal hydroxyl groups than the higher Mw EPS (Andrew and Jayaraman, 2020). In addition, lower Mw has the potential to release a greater number of protons when interacting with free radicals (Andrew and Jayaraman, 2020). Therefore, the antioxidant capacity increased with a decrease in Mw.

### 3.4 Elemental composition and chemical composition analysis of EPS

The basic elements constituting HD-01 EPS were C (40.97% ± 0.01%), H (6.88% ± 0.04%), N (2.86% ± 0.03%), and S (0.57% ± 0.02%). The chemical composition of HD-01 EPS was calculated by the standard curves of total sugar, protein, and sulfate groups. The results showed that the HD-01 EPS contained higher total sugar (77.77% ± 0.23%). The total sugar contents of *S. cerevisiae* EPS (61.79%) (Amer and Mubarak, 2013) and *P. flavescens* EPS (87%) (Oluwa, 2020) were also higher, which further implied that the biological activity of HD-01 EPS was mainly determined by glycosyl groups. There was a small amount of sulfate groups (0.36% ± 0.01%) in HD-01 EPS, which was closely related to antiviral and antitumor capacities (Anastyuk et al., 2017).

### 3.5 Microscopic morphology characterization

**FIGURE 3:**
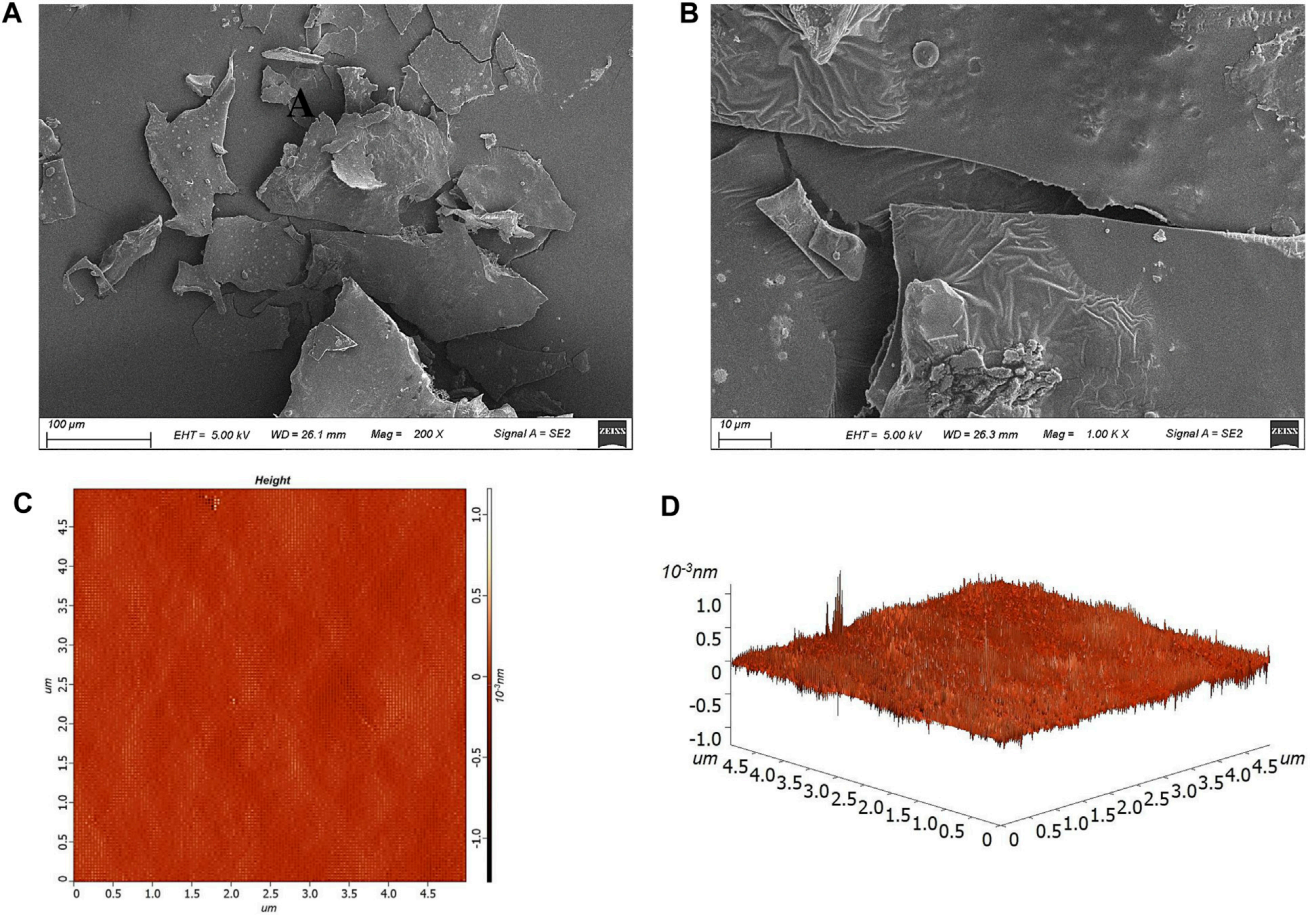
Microscopic morphology of *S. cerevisiae* HD-01 EPS with SEM [**(A)** ×200, **(B)** ×1,000] and AFM image [**(C)** plane view; **(D)** stereo view].

As shown in Figures 3A, B, the HD-01 EPS exhibited smooth, shiny, and compact sheet-like structures. *Paenibacillus polymyxa* 92 EPS was smooth on the surface and exhibited as a compact structure, which is consistent with this study (Grinev et al., 2020). Different surface structures of EPS resulted in various functional characteristics. The compact sheet structure endowed EPS with mechanical stability, which was conducive to the application in the pharmaceutical fields (Kanamarlapudi and Muddada, 2017). However, porous EPS had excellent properties of high viscosity and hydrophilia, making it possible to be used as a gelling agent or thickening agent (Feng et al., 2018).

AFM could directly characterize the topographic features of microbial EPS (Ahmed et al., 2013). As shown in Figures 3C, D, the spike-like structure with the height of 213.8 nm and average roughness of 13.8 nm was observed, and it presented a compact arrangement and a mesh-like configuration, which was in accordance with that of *Streptococcus thermophilus* CC30 EPS (Kanamarlapudi and Muddada, 2017). Studies have shown that EPS with a spike structure had strong hydrophilia and could be used as a thickener (Feng et al., 2019).

### 3.6 Structural properties

#### 3.6.1 XRD spectroscopy analysis

XRD results of HD-01 EPS are shown in Figure 2D. A diffraction peak appeared when 2θ° was 20, indicating that the HD-01 EPS was an amorphous non-crystalline substance, consistent with *Weissella confusa* XG-3 (Zhao et al., 2021). XRD results of *Lipomyces starkeyi* VIT-MN03 EPS showed it was a partially crystalline substance (Ragavan and Das, 2019), which is inconsistent with this study.

#### 3.6.2 FT-IR spectroscopy analysis

As shown in Figure 2E, the characteristic absorption peak of polysaccharides, observed at 3,408 cm^−1^, was indicative of the O–H bond’s stretching vibration (Seveiri et al., 2020). The C–H stretching vibration was observed at 2931.88 cm^−1^ (Ma et al., 2017). The existence of the C = O bond was supported by the peak at 1,657.22 cm^−1^. Peaks at 1,535.00 cm^−1^ and 1,411.70 cm^−1^ indicated the stretching vibrations of -CH_3_ and the carboxyl groups, respectively. The peaks at 1,254.65 cm^−1^, 1056.60 cm^−1^, 911.58 cm^−1^, and 813.61 cm^−1^ indicated the existence of the pyran ring configuration, *α*-1,6 glycosidic bonds, *β*-glycosidic bonds, and *α*-mannose, respectively, in EPS (Du et al., 2017; Ramirez, 2016; Wang et al., 2019b).

#### 3.6.3 Glycosidic bonding analysis

As shown in Table 1, HD-01 EPS was composed of glucose (29%) linked by 1-, 1, 4-, 1, 2, 3, 6-aglycosidic bonds and mannose (69%) linked by 1-, 1,2-, 1, 6-aglycosidic bonds, which is consistent with the results of monosaccharide composition. Based on the molar percentage of each residue, it can be inferred that the backbone was composed of 1-Man*p*. It was reported that *P. flavescens* EPS was mainly composed of (1→3)-pyranomanose and a small amount of (1→)-xylopyranose, (1→)-pyranomanose, and (1→)-glucopyranose (Oluwa, 2020). *Trichosporon asahii* EPS was composed of *α*-(1→3)-pyranomanose chain, *β*-(1→2)-D-pyran gluconic acid side chain, and xylopyranose assembled by *β*-(1→4) or *β*-(1→2) bonds (Fonseca et al., 2009). All these studies have confirmed that most yeast EPSs were heteropolysaccharides composed of mannose and glucose residues.

**TABLE 1:** Glycosidic bond connection mode of *S. cerevisiae* HD-01 EPS.

PMAA	Connection type	mol (%)	Fragment ion peak (m/z)
2,3,4,6-Me_4_-Glc*p*	1-	3.3	101,117,129,145,161,205
2,3,6-Me_3_-Glcp	1,4-	1.8	87,101,117,129,161,191,206,233
4-Me-Glc*p*	1,2,3,6-	24.3	87,101,129,189,262
2,3,4,6-Me_4_-Man*p*	1-	38.3	87,102,129,145,162,205
3,4,6-Me_3_-Man*p*	1,2-	17.5	87,129,161,189
2,3,4-Me_3_-Man*p*	1,6	14.8	102,118,129,161,189

**TABLE 2:** ^1^H and^13^C chemical shifts (ppm) of *S. cerevisiae* HD-01 EPS.

Sugar type		H1/C1	H2/C2	H3/C3	H4/C4	H5/C5	H6/C6
A *α*-1- mannose	H C	5.10 103.18	4.01 69.98	3.85 70.18	3.66 66.58	3.73 73.25	3.96 60.98
B *α*-1,2- mannose	H C	5.31 100.47	4.10 78.37	3.87 70.35	3.72 66.50	3.72 73.18	3.73 60.92
C *α*-1,6- mannose	H C	5.13 98.23	3.97 70.6	4.19 68.0	4.07 68.8	3.39 72.0	3.52 72.8
D *α*-1- glucose	H C	5.06 102.10	3.19 73.4	4.09 74.29	3.57 73.72	4.01 76.56	3.62 62.93
E *α*-1,2,3,6- glucose	H C	5.16 102.10	3.42 79.2	4.03 78.34	3.64 72.69	3.91 76.12	4.12 70.23
F *β*-1,4- glucose	H C	4.92 99.30	3.63 73.03	4.52 74.09	3.93 79.30	4.77 76.13	4.19 61.3

#### 3.6.4 NMR spectroscopy analysis

**FIGURE 4:**
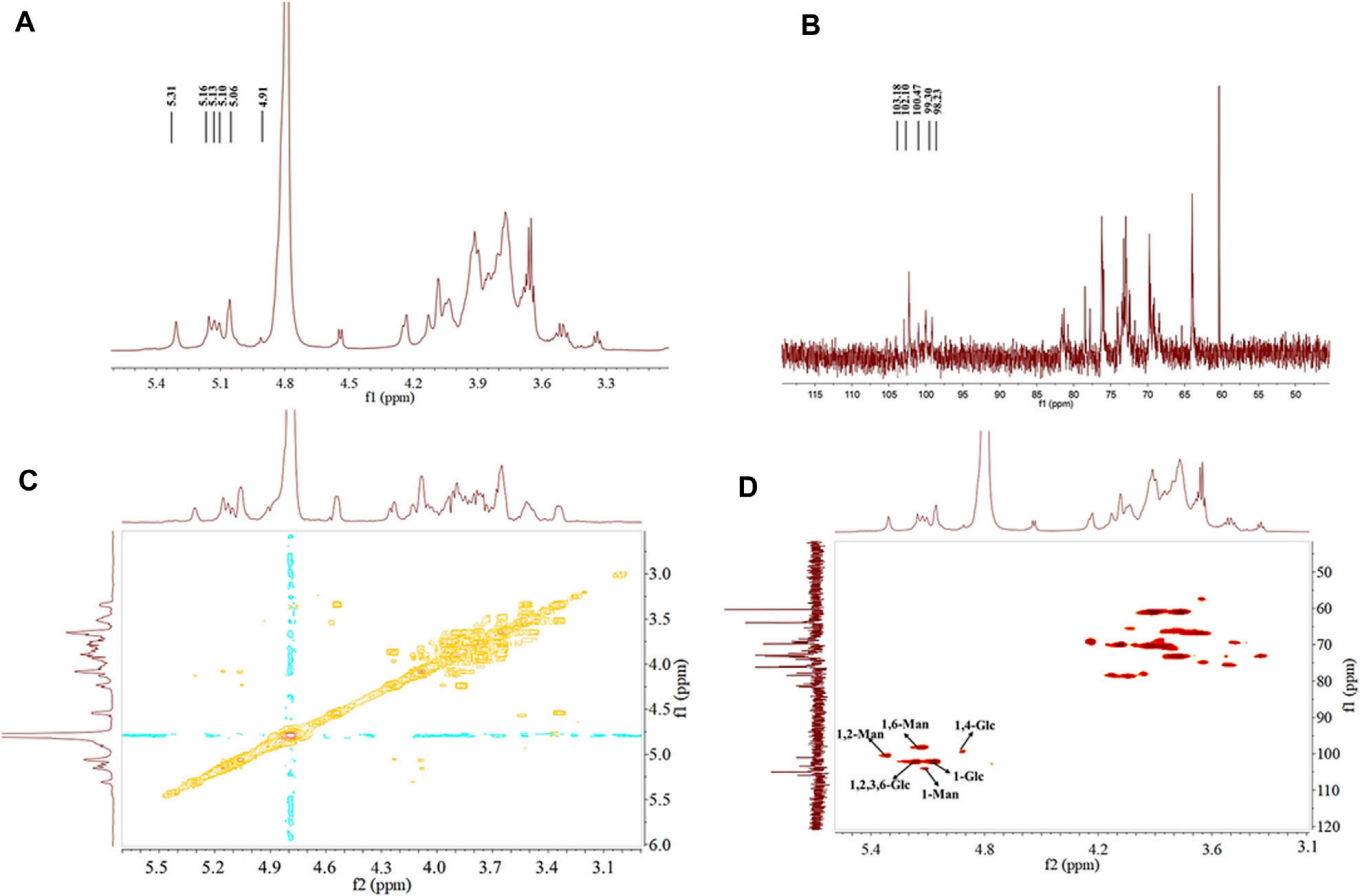
^1^H-NMR **(A)**, ^13^C-NMR **(B)**, ^1^H-^1^H COSY **(C)**, and ^1^H-^13^C HSQC **(D)** spectra of *S. cerevisiae* HD-01 EPS.

NMR was used in analyzing the configuration of glycosidic bonds in EPSs. The signal peak of EPS was mainly in the range of 3.0–5.5 ppm (Xu et al., 2017). In general, H1 proton displacement of the *α*-configuration exceeded 5.0 ppm, whereas that of the *β*-configuration was below 5.0 ppm (Sran et al., 2019). Six signal peaks appeared in the anomeric proton regions (Figure 4A) and were defined as A, B, C, D, E, and F (δ5.10, 5.31, 5.13, 5.06, 5.16, and 4.91, respectively) according to the sugar types, which revealed that HD-01 EPS contains both *α*- and *β*-glycosides. Combined with the methylation results, A, B, and C were attributed to *α*-mannose residues. D, E, and F were attributed to *α*- and *β*-glucose residues. ^13^C NMR could be used to identify anomeric carbon (Figure 4B). The peak at δ60-85 ppm in ^13^C NMR proved the presence of pyran ring, and there was no peak at δ90 ppm, which indicated the absence of a furan ring (Vinogradov et al., 1998). Xu et al. (2019) found the *Bacillus licheniformis* EPS had *α*-pyran and *β*-pyran configuration, consistent with HD-01 EPS. The hydrocarbon relationships of the residues (A, B, C, D, E, and F) were analyzed by COSY and HSQC, which were better than those ^1^D NMR methods in analyzing overlapping signals. As shown in Figures 4C, D and Table 1, for residue A, proton chemical shifts of H1–H6 (δ5.10, 4.01, 3.85, 3.66, 3.73, and 3.96 ppm) and chemical shifts of C1–C6 (δ103.18, 69.98, 70.18, 66.58, 73.25, and 60.98 ppm) were determined. In contrast to the standard methyl glycoside, the residue A was not substituted and belonged to the *α*-1 glycosidic bond, implying that residue A was *α*-1-mannose. For residue B, the proton chemical shifts of H1–H6 were δ5.31, 4.10, 3.87, 3.72, 3.72, and 3.73 ppm, and the chemical shifts of C1–C6 were δ100.47, 78.37, 70.35, 66.50, 73.18, and 60.92 ppm. C2 was the glycosylation site. Compared with residue A, the chemical shift was shifted to the lower field, indicating that residue B was substituted at C2 and belonged to the *α*-1,2 glycosidic bond. Therefore, residue B was *α*-1, 2-mannose. Similarly, residue C was *α*-1, 6-mannose. For residue D (H1–H6: δ5.06, 3.19, 4.09, 3.57, 4.01, and 3.62 ppm; C1–C6: δ102.10, 73.4, 74.29, 73.72, 76.56, and 62.93 ppm), it was not substituted and belonged to the *α*-1 glycosidic bond, implying that residue D was *α*-1-glucose. For residue E, the proton chemical shifts of H1–H6 were δ5.16, 3.42, 4.03, 3.64, 3.91, and 4.12 ppm and the chemical shifts of C1–C6 were δ102.10, 79.2, 78.34, 72.69, 76.12, and 70.23 ppm. Compared with residue D, the chemical shifts (C2/C3/C6) were shifted to the lower field, indicating that residue E was substituted at C2/C3/C6 and belonged to the *α-*1,2,3,6 glycosidic bond. Therefore, residue E was *α*-1,2,3,6-glucose. Similarly, for F (H1–H6: 4.92, 3.63, 4.52, 3.93, 4.77, and 4.19; C1–C6: 99.30, 73.03, 74.09, 79.30, 76.30.76.13, and 61.3), the chemical shift (C4) was shifted to the lower field, indicating that residue F was *β*-1, 4-glucose.

All results of HD-01 EPS revealed the HD-01 EPS was a HePS composed of 38.3% *α*-1, 17.5% *α*-1,2, 14.8% *α*-1, 6-linked mannose and 24.3% *α*-1, 2, 3, 6, 3.3% *α*-1, 1.8% *β*-1, 4-linked glucose.

#### 3.6.5 N-glycosylation and O-glycosylation configuration analysis

**FIGURE 5:**
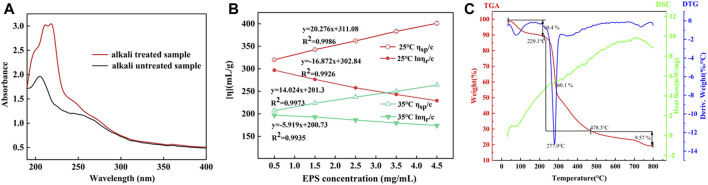
Absorbance of alkali-treated and alkali-untreated *S. cerevisiae* HD-01 EPS **(A)**. Intrinsic viscosity of *S. cerevisiae* HD-01 EPS at 25°C and 35°C **(B)**. DSC, TGA, and DTA curves of *S. cerevisiae* HD-01 EPS **(C)**.

The linkage types of glycopeptide chains are mainly divided into two types (N- and O-bond linkage). N-glycosylation occurred in mannose (Wang et al., 2014). The presence of mannose could preliminarily infer there was N-glycosylation in HD-01 EPS. *β*-elimination was used to analyze the existence of O-glycosylation. The absorbance of alkali-treated HD-01 EPS at 240 nm was significantly increased compared with that of alkali-untreated polysaccharides, indicating that *β*-elimination occurred and O-glycosylation existed in HD-01 EPS (Figure 5A). The HD-01 EPS was different from most EPSs in the way of glycopeptide chain connection, such as *Leuconostoc mesenteroides* DRP105 EPS (Xing et al., 2018), *Gracilariopsis lemaneiformis* EPS (Shi et al., 2017), and pumpkin seeds EPS (Wang et al., 2017a) only containing O-glycosylation.

### 3.7 Determination of intrinsic viscosity

The intrinsic viscosity [*η*] of polysaccharides mainly depends on the solvent properties and Mw of the polysaccharide (Jiang et al., 2020). The intrinsic viscosity of HD-01 EPS at 25°C and 35°C was measured using the Ubbelohde viscometer, and the results are shown in Figure 5B. Through intersection of the intercept at the Y-axis by calculating the Huggins and Kraemer equation line extrapolation, the intrinsic viscosity at 25°C and 35°C was 219.52 mL/g and 192.63 mL/g, respectively. The HD-01 EPS had lower viscosity than the reported EPS of other strains, such as *W. confusa* XG-3 EPS (409.7 mL/g) (Zhao et al., 2020) and *Astragalus gombo* galactomannan (860 mL/g) (Chouana et al., 2017). There were negative correlations between intrinsic viscosity and temperature, which might be because viscosity was closely related to the Mw. EPS with a smaller Mw was easier to dissolve, and the viscosity decreased when heated (Du et al., 2017). Therefore, the structure was more stable with lower viscosity and smaller Mw.

### 3.8 Thermal properties

The thermal properties of EPS are the key to determine its potential industrial application (Lakra et al., 2020). The thermogravimetric dynamic of HD-01 EPS was determined (Figure 5C). The TGA curve showed that gelation and swelling occurred with the increase in temperature, followed by dehydration and cracking. In the first stage (40°C–209°C), 10.4% was lost with the loss of free water, indicating that carboxyl groups in HD-01 EPS were bound to water (Wang et al., 2015). In the second stage (229.1°C–475.6°C), the heat was lost (60.1%) due to the degradation of EPS. The third stage occurred at 478.3°C, resulting in mass loss (9.57%) (Zhao et al., 2021) due to the degradation of other inorganic materials.

The DTG curve showed a distinct peak at 277.9°C, representing the degradation temperature (Td) of HD-01 EPS. Td of HD-01 EPS was higher than that of *Leuconostoc lactis* KC117496 (241.14°C) (Saravanan and Shetty, 2016) and *Halomonas* SP. AAD6 (253°C) (Annarita et al., 2009). The different thermostability of EPS might be related to its monosaccharide composition. In the DSC curve, a thermal absorption peak at 84°C was observed due to the dehydration of the sample. This was followed by subsequent melting peaks and crystallization peaks, possibly indicating the reformation of refractory components in the sample post decomposition (Aburas et al., 2020). The results implied HD-01 EPS had great thermostability and could be used in various fields.

### 3.9 WSI and WHC of EPS

The WSI and WHC of HD-01 EPS were 89.2% ± 0.75% and 349.47% ± 0.2%, respectively, demonstrating EPS had great water solubility and WHC. Wang et al. (2010) found that the WSI of EPS was related to the composition of monosaccharides. WSI of EPS-containing glucose was higher, while WHC was related to a high polymer chain that could retain a large amount of water with hydrogen bonding, making it be used as a biological surfactant and stabilizer in fermented food.

### 3.10 Water contact angle analysis

**FIGURE 6:**
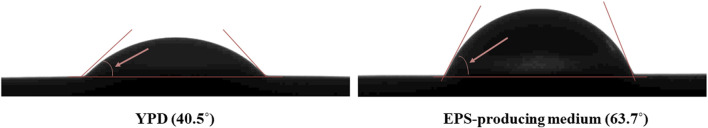
Contact angle results of *S. cerevisiae* HD-01.

The contact angles of HD-01 in YPD and the EPS-producing medium are shown in Figure 6. The contact angle in the EPS-producing medium containing sucrose (63.7°) was larger than that in the YPD medium (40.5°), proving that the sucrose could induce HD-01 to produce EPS, and with the formation of HD-01 EPS, the surface hydrophobicity was enhanced. Research findings indicated that the surface hydrophobicity of the strain was found to be intricately linked to the structure of the surface protein (Vadillo-Rodriguez et al., 2004), which was consistent with the results of LAB-producing EPS. The contact angle of *L. plantarum* 23 was lower in the MRS medium compared to that in MRS-S (5% sucrose) medium, implying that sucrose promoted the production of EPS (Yu et al., 2018).

### 3.11 Growth characteristics of probiotic bacteria

**FIGURE 7:**
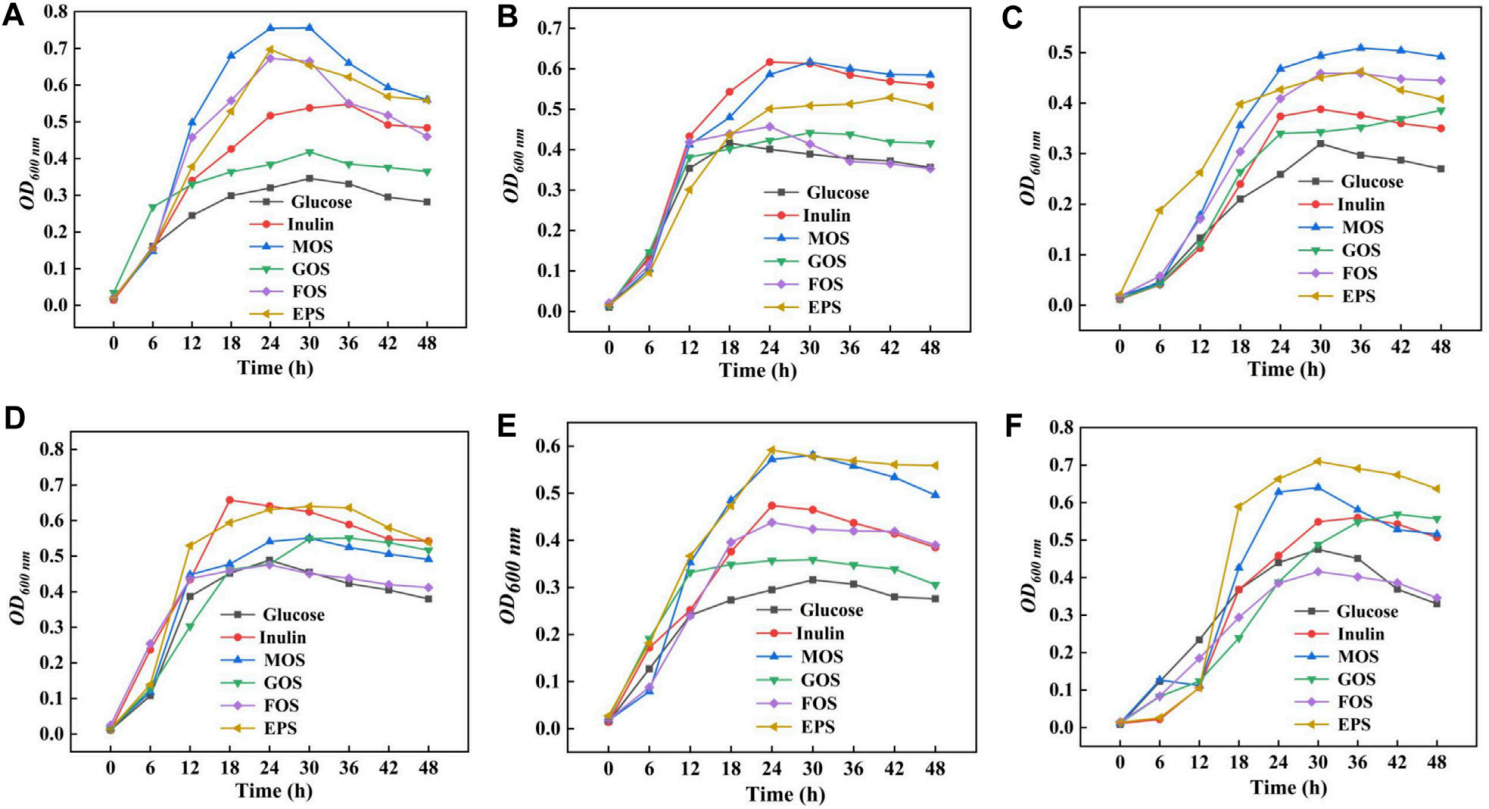
Proliferation effect of *S. cerevisiae* HD-01 EPS and commercial prebiotics [inulin, mannooligosaccharide (MOS), galactose oligosaccharide (GOS), and fructose oligosaccharide (FOS)] on probiotics [**(A)** *L. plantarum* subsp. CICC 6076, **(B)** *B. adolescentis* QY03, **(C)** *L. casei* QY02, **(D)** *L. delbrueckii* subsp. CICC 6077, **(E)** *L. paracei* HD1.7, and **(F)** *L. acidophilus* QY01].

In order to detect the probiotic properties of HD-01 EPS, six probiotics were cultured in the MRS medium containing prebiotics and HD-01 EPS, respectively. The growth curve of probiotics was obtained in 48 h. Compared with the control groups, four prebiotics and HD-01 EPS showed different effects on promoting the six probiotics (Figure 7). The promotion effects of HD-01 EPS on *L. paracei* HD1.7 and *L. acidophilus* QY01 were significant, and the strains grew best at 24 h, indicating that HD-01 EPS had potential as a prebiotic (Heperkan et al., 2020).

### 3.12 *In vitro* antioxidant activity assay of *S. cerevisiae* HD-01 EPS

#### 3.12.1 DPPH radical scavenging assay

**FIGURE 8:**
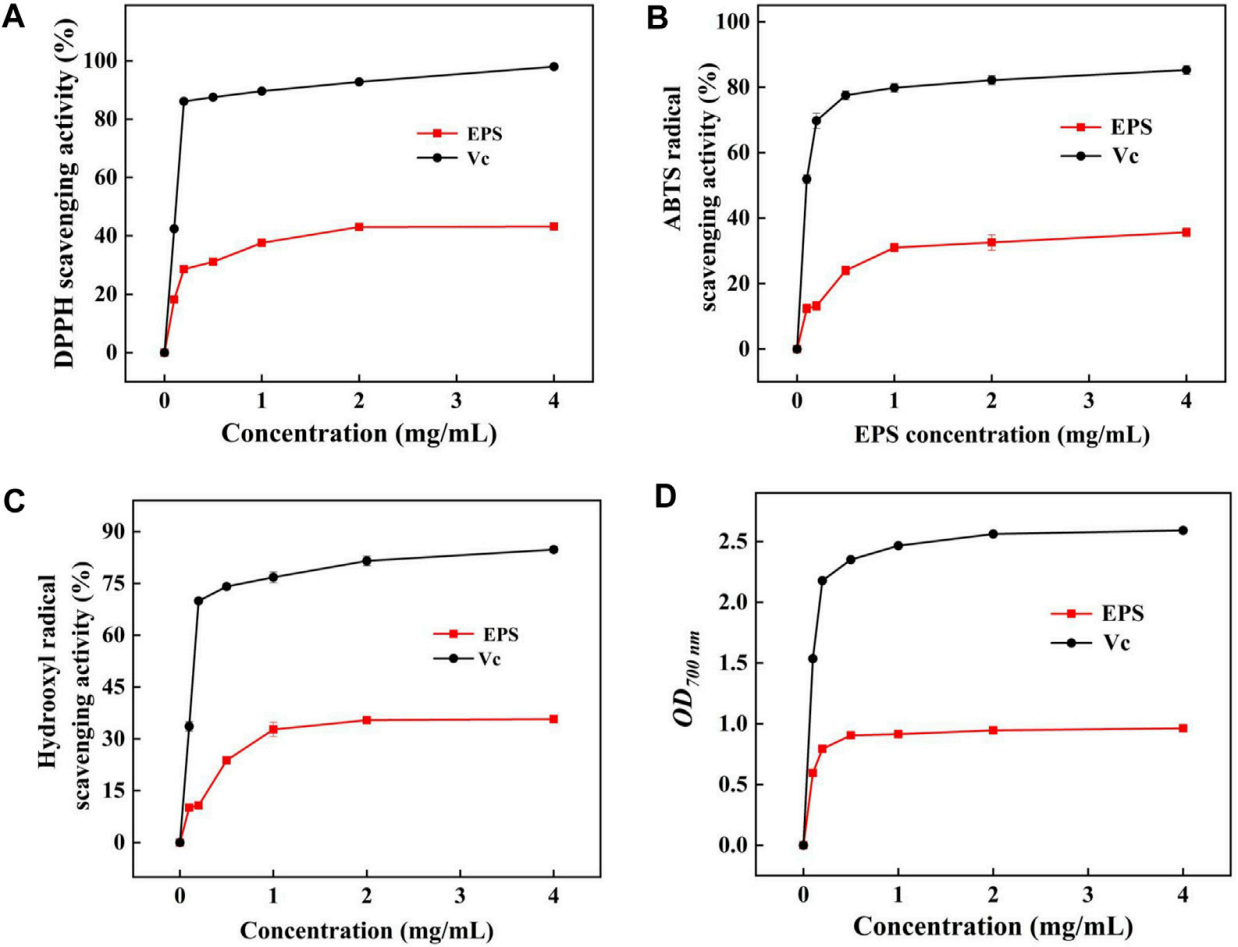
Scavenging activity of DPPH **(A)**, ABTS **(B)**, and hydroxyl radicals **(C)** and reducing power **(D)** of *S. cerevisiae* HD-01 EPS.

DPPH radical is a kind of relatively stable synthetic free radical that has strong scavenging ability attributed to the structure of EPS (Du et al., 2019). The DPPH radical scavenging ability gradually increased in a dose-dependent manner (Figure 8A). The DPPH radical scavenging ability of HD-01 EPS was 37.59% ± 0.67% at 1.0 mg/mL, which was higher than *R. mucilaginosa* CICC 33013 EPS (25.05% ± 1.60%) (Ma et al., 2017). When the concentration of HD-01 EPS was up to 4 mg/mL, the strongest scavenging DPPH activity (43.17% ± 0.47%) was observed. Functional groups such as -O-, C=O, and -OH can donate electrons or hydrogen to DPPH radicals, leading to a notable decrease in free radicals and demonstrating impressive functional properties (Wang et al., 2019a).

#### 3.12.2 ABTS radical scavenging assay

ABTS radicals could react with K_2_SO_3_, making the solution turn green and having the maximum absorbance at 734 nm (Rani et al., 2017). As shown in Figure 8B, the scavenging ability of HD-01 EPS increased with concentration, but it was still lower than the scavenging ability of Vc at equivalent concentrations. The ABTS radical scavenging ability reached the highest (35.65% ± 1.34%) when the concentration of HD-01 EPS was 4.0 mg/mL, which was similar with that of the EPS from *S. cerevisiae* Y3 [24.8% ± 1.34% (Liu et al., 2021)]. The ability to scavenge ABTS radicals is different among strains, which attributes to the composition, protein content, and functional groups of EPS.

#### 3.12.3 Hydroxyl radical scavenging assay

Hydroxyl radical has strong oxidability and could cause harm to biological tissue. The hydroxyl radical scavenging ability was gradually enhanced with the increase in HD-01 EPS and reached the highest (35.71% ± 0.89%) value at the concentration of 4 mg/mL (Figure 8C). The result was in accordance with that of *R. babjevae* EPS [65% (Seveiri et al., 2020)]. Studies have shown that the hydroxyl radical scavenging activity was closely related to the monosaccharide composition of EPS, and the presence of mannose made EPS have good antioxidant properties (Oluwa, 2020), which is consistent with the HPLC result of HD-01 EPS.

#### 3.12.4 Reducing power assay

As shown in Figure 8D, the reducing power of HD-01 EPS reached the highest (0.96 ± 0.09) at 700 nm at the concentration of 4 mg/mL, which was higher than that of *Leuconostoc citreum* B-2 EPS (0.28 ± 0.01) (Wang et al., 2019b). All results proved that HD-01 EPS had the potential to be utilized as an antioxidant and applied in the food and medical fields.

### 3.13 Effects of EPS on the viability of RAW264.7 cells

**FIGURE 9:**
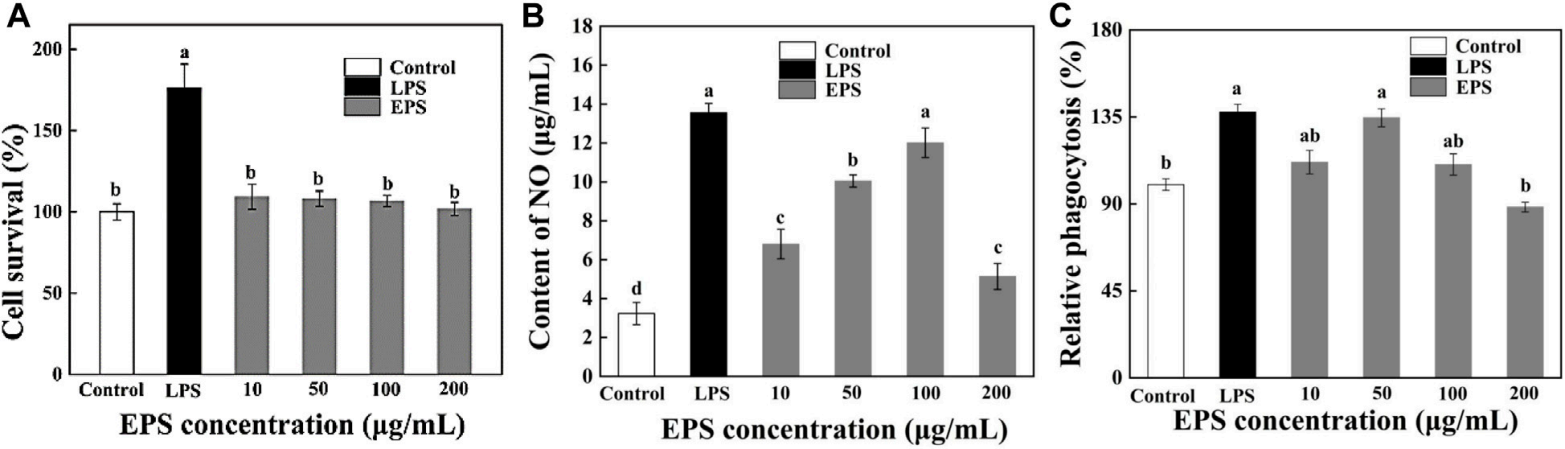
Effects of *S. cerevisiae* EPS on the survival ratio **(A)**, NO secretion **(B)**, and phagocytosis **(C)** of RAW264.7 cells. Note: different letters indicate significant differences (*p* < 0.05).

The cell viability of HD-01 EPS positively correlated with *OD*_*570 nm*_ on RAW264.7 cells was detected by MTT assay. Figure 9A revealed the cytotoxic effects with different concentrations of HD-01 EPS on RAW264.7 cells. Compared with the control group, the cell survival rate reached 165% ± 12.35% with LPS, showing that there was a strong proliferation effect on RAW264.7 cells (*p* < 0.05). The survival rate reached 95% when the HD-01 EPS concentration was 10–200 μg/mL, which showed that HD-01 EPS had no toxic effect on cells and no proliferation effect at 10–200 μg/mL, indicating that HD-01 EPS used in this experiment was in the safe concentration range. The toxicity analysis of *Cyclocarya paliurus* polysaccharide on RAW264.7 cells found that the polysaccharide had no toxic effect on the cells at the concentration of 25–200 μg/mL (Zhang et al., 2021), which is consistent with this study.

### 3.14 Effects of EPS on NO secretion

It is reported that macrophages secrete NO after being activated, which could help exert immune regulation (Zhang et al., 2021). Compared with the blank control group, the NO secretion of RAW264.7 cells was significantly increased with HD-01 EPS in the range of 10–100 μg/mL and reached the highest at 100 μg/mL (Figure 9B), indicating that HD-01 EPS could activate the NO secretion of macrophage RAW264.7 at a certain concentration range. Xu et al. (2012) found that *Lentinula edodes* L2 EPS could significantly promote the secretion of NO, which is consistent with this study.

### 3.15 Effects of EPS on phagocytosis of RAW264.7 cells

The phagocytic ability of cells is the key to assay cell activity. In this research, the effect of HD-01 EPS on the phagocytosis of RAW264.7 was assessed via the neutral red method (Chen et al., 2016). The phagocytosis of HD-01 EPS on RAW264.7 cells increased at the concentration of 10–50 μg/mL and reached the highest (134.6% ± 4.7%) at 50 μg/mL (Figure 9C), indicating that HD-01 EPS could enhance phagocytosis and improve immunity. *Poria cocos* SGRP1 EPS (Wang et al., 2017b) and *Cordyceps militaris* PLCM EPS (Lee et al., 2015) could also significantly promote the phagocytosis of macrophages. Studies have shown that glucose, as well as mannose, had high anti-tumor activity (Ai-Lati et al., 2017). The HPLC result revealed that HD-01 EPS was mainly composed of mannose and glucose, which was in accordance with this result.

### 3.16 Effect of HD-01 EPS on production of IL-6, IL-8, IL-1*β*, IL-10, MCP-1, and TNF-*α*

**FIGURE 10:**
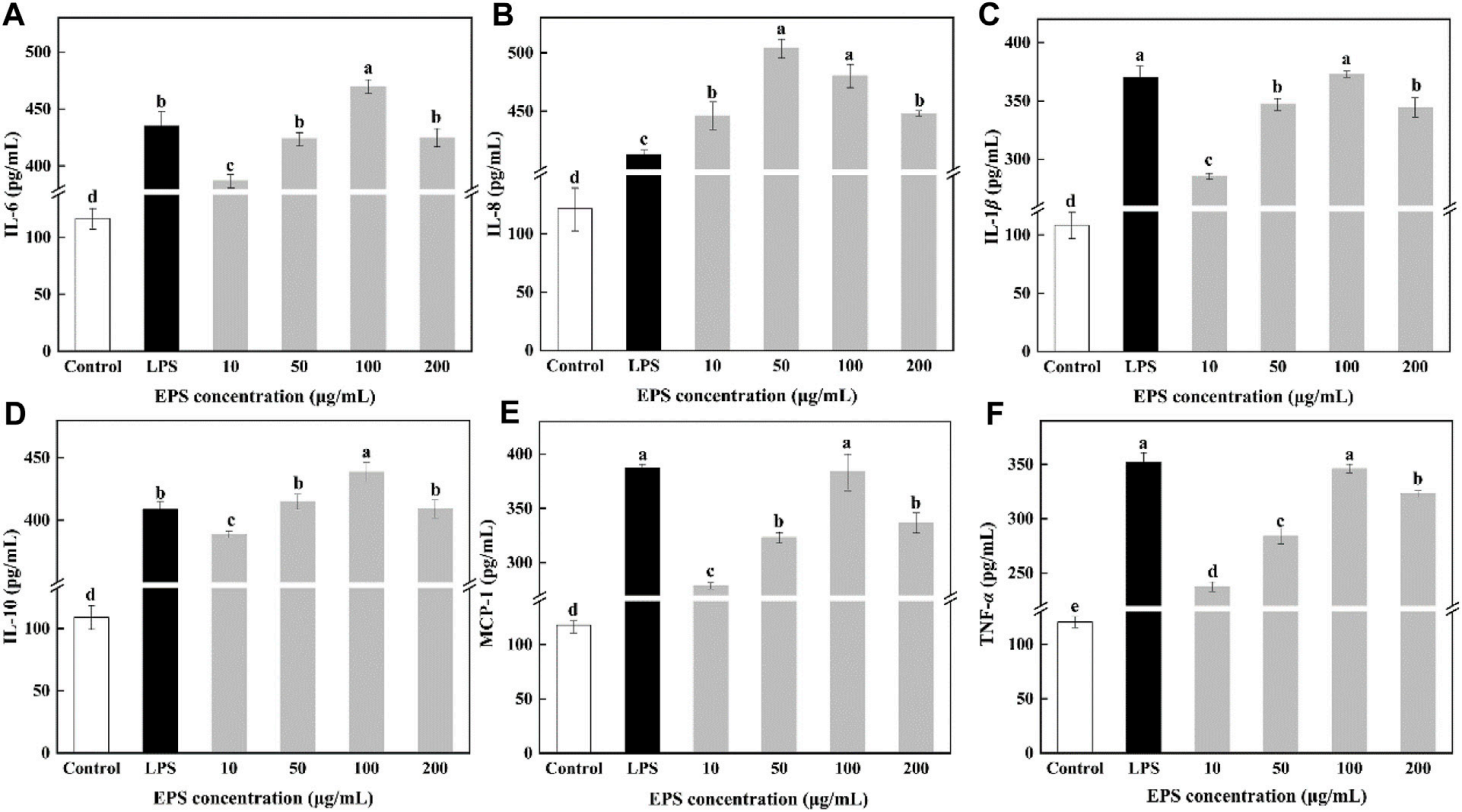
Effect of *S. cerevisiae* HD-01 EPS on IL-6 **(A)**, IL-8 **(B)**, IL-1β **(C)**, IL-10 **(D)**, MCP-1 **(E)** and TNF-α **(F)** secretion of RAW264.7 cells. Note: Different letters indicate significant differences (*p* < 0.05).

As an important factor of immune regulation, cytokines play a vital role in immunomodulation (Pontigo and Vargas-Chacoff, 2020). IL-6 secreted by activated macrophages plays a role in the development of a range of inflammatory and immune-related disorders (Cai et al., 2021). IL-8 could induce cell proliferation. IL-1*β* could promote cell proliferation and participate in the inflammatory response. TNF-α is a pro-inflammatory cytokine, which can participate in the inflammatory response and the expression of immunomodulatory mediators (Zhang et al., 2021). Macrophages could stimulate the secretion of those cytokine to exert immune responses. As shown in Figure 10, different concentrations of HD-01 EPS significantly increased the secretion of IL-6, IL-8, IL-1*β*, IL-10, MCP-1, and TNF-*α* compared with the control groups, indicating that HD-01 EPS could significantly activate macrophages to release cytokines. Cytokines (IL-6, IL-10, IL-1*β*, MCP-1, and TNF-*α*) reached the highest at the concentration of 100 μg/mL of HD-01 EPS, while IL-8 reached the highest at 50 μg/mL. The peak values of IL-6, IL-8, and IL-10 were significantly higher than that of the LPS-positive control groups. You et al. (2020) found that *Lactobacillus pentosus* R-17-EPS significantly induced the secretion of TNF-*α*, IL-1*β*, and IL-6, which was in accordance with this study. Similarly, Wang et al. (2020) found that EPS103 from *L. plantarum* JLAU103 enhanced the phagocytic activity of RAW264.7 macrophages and promoted the secretion of IL-6 and TNF-*α*. All results implied that HD-01 EPS enhanced the phagocytic capacity of RAW264.7 cells and the immune response of macrophages to tumor cells, and it possessed the potential be applied in vaccine development and tumor immunotherapy in the future.

## 4 Conclusion

An EPS-producing yeast was identified as *S. cerevisiae*. The HD-01 EPS was mainly composed of *α*-1 (38.3%), *α*-1,2 (17.5%), *α*-1, 6 (14.8%)-linked mannopyranose and *α*-1, 2, 3, 6 (24.3%), *α*-1 (3.3%), *β*-1, 4 (1.8%)-linked glucopyranose. It contained a high content of carbohydrates and a small amount of N and S, indicating that sulfation modification existed in it. Microscopically, the HD-01 EPS had a smooth surface, compact flake structure, and network configuration, contributing to the preparation as a plastic material. Similar to other food-grade bacterial EPS, the HD-01 EPS exhibited great WHS, WSI, probiotic, and antioxidant capacities, making it possible to use it as a stabilizer, moisturizer, and prebiotics in the food field. Meanwhile, HD-01 EPS could promote the proliferation of RAW264.7 cells, the release of cytokines, and enhance the phagocytosis of RAW264.7 cells, indicating that it had a positive effect on the immunomodulatory properties of macrophages and had the potential to be used as vaccine adjuvants in immunomodulation. All these results suggested that the HD-01 EPS had a potential to be applied in the food and pharmaceutical industries and laid the foundation for further studies on its anti-inflammatory and anti-tumor effects.

## Data Availability

The datasets presented in this study can be found in online repositories. The names of the repository/repositories and accession number(s) can be found in the article/supplementary material.
